# Comprehensive assessment of body mass index effects on short-term and long-term outcomes in laparoscopic gastrectomy for gastric cancer: a retrospective study

**DOI:** 10.1038/s41598-024-64459-w

**Published:** 2024-06-15

**Authors:** Hai Hu, Lili Hu, Kun Li, QiHua Jiang, JunTao Tan, ZiQing Deng

**Affiliations:** 1 Department of General Surgery, Third Hospital of Nanchang, No. 2, Xiangshan South Road, Xihu District, Nanchang City, Jiangxi Province China; 2 Department of Pediatrics, Third Hospital of Nanchang, No. 2, Xiangshan South Road, Xi hu District, Nanchang city, China; 3 Department of Breast Surgery, Third Hospital of Nanchang, No. 2, Xiangshan South Road, Xi Hu District, Nanchang City, Jiangxi Province China; 4 Jiangxi Province Key Laboratory of Breast Diseases, Third Hospital of Nanchang, No. 1268, Jiuzhou Street, Chaoyang New Town, Xihu District, Nanchang City, China

**Keywords:** Gastric cancer, Laparoscopic gastrectomy, Body Mass Index, Surgical outcomes, Postoperative complications, Survival analysis, Gastrointestinal cancer, Cancer, Risk factors

## Abstract

To examine the influence of Body Mass Index (BMI) on laparoscopic gastrectomy (LG) short-term and long-term outcomes for gastric cancer. A retrospective analysis was conducted on gastric cancer patients undergoing LG at the Third Hospital of Nanchang City from January 2013 to January 2022. Based on WHO BMI standards, patients were categorized into normal weight, overweight, and obese groups. Factors such as operative time, intraoperative blood loss, postoperative complications, and overall survival were assessed. Across different BMI groups, it was found that an increase in BMI was associated with longer operative times (average times: 206.22 min for normal weight, 231.32 min for overweight, and 246.78 min for obese), with no significant differences noted in intraoperative blood loss, postoperative complications, or long-term survival among the groups. The impact of BMI on long-term survival following LG for gastric cancer was found to be insignificant, with no notable differences in survival outcome between different BMI groups. Although higher BMI is associated with increased operative time in LG for gastric cancer, it does not significantly affect intraoperative blood loss, postoperative complications, recovery, or long-term survival. LG is a feasible treatment choice for obese patients with gastric cancer.

## Introduction

Despite significant advancements in the treatment of gastric cancer increasing patient survival outcome, it still ranks as the third most common cause of death from cancer globally^1^. For patients with advanced-stage gastric cancer, curative surgery combined with thorough lymph node dissection remains the only possible method for achieving a cure^2,3^. In comparison to traditional open surgery, laparoscopic gastrectomy (LG) for gastric cancer is gradually becoming an alternative treatment option due to its advantages, including smaller surgical trauma, reduced intraoperative bleeding, lower postoperative complications, and faster recovery^4–6^. However, this approach places higher demands on the surgical skills and techniques of surgeons, leading to a series of challenges.

Obesity is viewed as one of the important worldwide public health challenges, with its incidence rising consistently across nearly all nations and having seen a twofold increase in 70 countries since 1980^7^. Consequently, surgeons are increasingly coming across a higher number of obese patients in their routine clinical work. Simultaneously, there appears to be a connection between obesity and an increased risk of gastric esophageal cancer^8,9^. The effect of Body Mass Index (BMI) on the operative time has been studied in various tumor surgeries, including colorectal surgery^10^, pancreaticoduodenectomy^11^, liver surgery^12^, and esophagectomy^13^. However, research results regarding whether BMI affects the operative time in LG for gastric cancer have been contradictory. While most studies suggest that obesity may increase surgical difficulty, such as prolonging surgical time, increasing intraoperative bleeding, and reducing the number of lymph node dissections^14–17^, there are also studies indicating that elevated BMI does not extend operative time or affect the thoroughness of lymph node removal and the occurrence of complications after surgery^18–20^. It is noteworthy that most studies from the Asian region adopt a threshold of 25 kg/m^2^ for obesity categorization, which is not consistent with the existing standards set by the World Health Organization (WHO) for defining obesity, adding complexity to the interpretation of these results^21^.

To address these uncertainties, we carried out a retrospective study aimed at evaluating the impact of different BMIs (overweight, obese) on the operative time in LG for gastric cancer, alongside other short-term surgical outcomes. Furthermore, we explored the potential effects of different BMIs on the long-term surgical outcomes.

## Materials and methods

### Subjects

**Figure 1:**
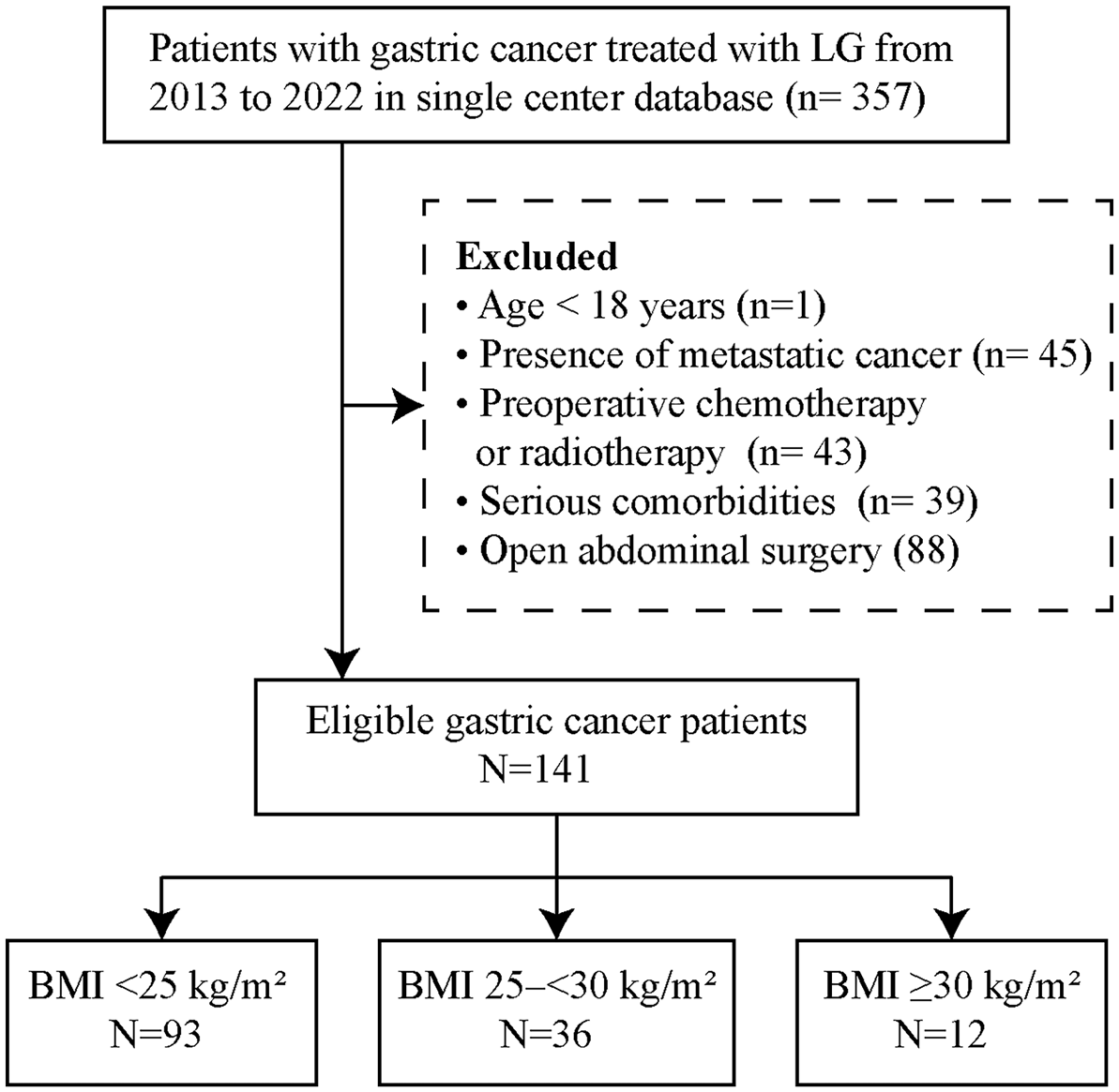
Flow chat of selecting patients in the study.

This study retrospectively analyzed patient data of those who underwent LG for gastric cancer at the Third Hospital in Nanchang from January 2013 to January 2022. Inclusion criteria were required to be older than 18 years and preoperative diagnosis of gastric cancer (clinical stages I–III). Criteria for exclusion included cases with metastatic cancer, preoperative chemotherapy or radiotherapy, as well as the presence of serious comorbidities such as severe cardiovascular diseases (e.g., congestive heart failure, unstable angina), advanced respiratory diseases (e.g., severe chronic obstructive pulmonary disease, interstitial lung disease), uncontrolled diabetes mellitus with complications, severe hepatic dysfunction, end-stage renal disease requiring dialysis, significant coagulopathies, or any other conditions determined by the attending physician to substantially increase perioperative risks. Additionally, patients with previous experience of open abdominal procedures were also excluded from the study. Study flow is illustrated in Fig. 1. Patients were grouped into three classifications following the World Health Organization (WHO) BMI criteria: within the normal weight range (BMI < 25 kg/m^2^), categorized as overweight (BMI 25–< 30 kg/m^2^), and identified as obese (BMI ≥ 30 kg/m^2^)^22^.

### Surgical procedures

All patients underwent surgery performed by a team of experienced surgeons following a standardized protocol for curative laparoscopic gastric cancer surgery, including total or partial gastrectomy along with necessary lymph node dissection^23,24^. All surgeries were performed by a team of experienced surgeons specialized in laparoscopic gastric cancer procedures. These surgeons underwent extensive training and accumulated significant expertise in performing laparoscopic surgeries.

### Data collection

Data including age, gender, BMI, comorbidities (such as diabetes, hypertension), operative time, blood loss during operation, hospitalization period, and complications within 30 days post-surgery was gathered from patient medical files. The operative time was measured from the moment the laparoscopy entered the abdominal cavity until the completion of skin suturing. Long-term surgical outcomes were mainly assessed by comparing survival analysis among various BMI groups.

### Follow-up

In the year following surgery, patients had quarterly check-ups, then every 6 months for the next 4 years. Routine follow-up assessments included blood tests, tumor marker screenings, gastroscopy, and abdominopelvic CTs. The follow-up period varied among the BMI categories: for normal weight patients, the median follow-up duration was 23.5 months (range 4.1–59.5 months); for overweight patients, it was 24.2 months (range 4.4–58.6 months); and for obese patients, it was 25.8 months (range 4.8–57.9 months). Long-term data on survival time were obtained through telephone interviews.

### Statistical analysis

IBM SPSS Statistics software (version 23.0) was used for statistical analysis. Continuous variables (mean ± SD) were compared using t-tests or Mann–Whitney U tests, while categorical data (% counts) were analyzed with chi-square or Fisher's exact tests (two-tailed). Survival curves were generated using Kaplan–Meier method and compared with log-rank tests, considering a P-value < 0.05 as statistically significant.

### Literature review

Our literature search was conducted on PubMed, focusing on the terms "gastric neoplasms," "gastric carcinoma," "gastric cancer," "laparoscopic surgery," "gastric resection," and "gastrectomy" to identify studies exploring the influence of BMI on LG outcomes in gastric cancer. Additionally, we manually reviewed references and articles to ensure a comprehensive inclusion of relevant studies. Inclusion criteria for our review were straightforward: studies needed to (1) investigate BMI's impact on LG in gastric cancer, and (2) be published in English for direct relevance to our study.

### Ethics statement

The study received ethical approval from Nanchang's Third Hospital Ethics Committee (Approval ID: K-lw2023006), and participants provided informed written consent in line with the Declaration of Helsinki.

## Results

### Patient characteristics

Based on the inclusion and exclusion criteria, a total of 141 patients were eligible. Among them, 93 patients had a BMI < 25 kg/m^2^, 36 patients had a BMI between 25 and < 30 kg/m^2^, and 12 patients had a BMI ≥ 30 kg/m^2^ (Fig. 1). Table 1 displays patient clinicopathological features, categorized by BMI (Normal weight, Overweight, Obese. BMI values significantly rose across groups, especially between normal weight and overweight/obese groups (P < 0.001 for both). Comorbidity analysis revealed significant variations in hypertension prevalence between obese and normal weight individuals (P < 0.05), though no substantial differences were found in other conditions like diabetes, cardiovascular, pulmonary, liver, and renal diseases among different BMI groups. Moreover, age, gender, tumor size, TNM staging, and surgical extension did not exhibit significant variations among the different BMI groups. These findings indicated that elevated BMI correlates with a greater occurrence of certain comorbidities, but had minimal impact on other clinicopathological characteristics.

**Table 1:** Comparison of the clinicopathological characteristics, stratified by BMI.

Variables	Normal weight (n = 93) N (%)	Overweight (n = 36) N (%)	Obese (n = 12) N (%)	P_1_ Normal versus overweight	P_2_ Normal versus obese
Age (y)	59.66 ± 11.54	60.32 ± 10.98	61.07 ± 11.75	0.768	0.692
Male	61 (65.6)	23 (63.9)	7 (58.3)	0.856	0.620
BMI (kg/m^2^)	22.34 ± 2.03	26.91 ± 1.58	31.9 ± 1.79	< 0.001	< 0.001
Comorbidity					
Hypertension	23 (24.7)	13 (36.1)	7 (58.3)	0.196	0.015
Diabetes	12 (12.9)	8 (22.2)	3 (25.0)	0.190	0.260
Cardiovascular disease	2 (2.2)	1 (2.8)	0 (0.0)	0.832	0.608
Pulmonary disease	5 (5.4)	1 (2.8)	1 (8.3)	0.530	0.678
Liver disease	2 (2.2)	1 (2.8)	1 (8.3)	0.832	0.226
Renal disease	1 (1.1)	0 (0.0)	0 (0.0)	0.532	0.718
Tumor size (cm)	2.88 ± 1.73	2.78 ± 1.85	2.71 ± 1.62	0.773	0.748
TNM stage				0.438	0.703
I	16 (17.2)	6 (16.7)	2 (16.7)		
II	21 (22.6)	12 (33.3)	4 (33.3)		
III	56 (60.2)	18 (50.0)	6 (50.0)		
Surgical extension				0.490	0.465
Distal gastrectomy	64 (68.8)	27 (75.0)	7 (58.3)		
Total gatrectomy	29 (31.2)	9 (25.0)	5 (41.7)		

Values shown are mean ± SD or n (%); BMI, body mass index.

### Intraoperative results, postoperative complications, and recovery

Table 2 revealed intraoperative results, postoperative complications, and recovery metrics across different BMI categories. These results indicated that the operative time significantly increased with the rise in BMI categories; Specifically, the mean overall operative time for normal weight, overweight, and obese individuals was 206.22 min, 231.32 min, and 246.78 min, respectively, with these differences being statistically significant (all P < 0.05). Further analysis revealed that for distal gastrectomy, the mean operative time was 186.24 ± 57.28 min for the normal weight group, 209.43 ± 61.35 min for the overweight group, and 224.35 ± 61.68 min for the obese group, with these differences also statistically significant (all P < 0.05). For total gastrectomy, the mean operative time was 226.57 ± 65.34 min for the normal weight group, 252.62 ± 69.42 min for the overweight group, and 268.35 ± 71.22 min for the obese group, with these differences being statistically significant as well (all P < 0.05). Nonetheless, no significant differences were observed among the three groups in terms of conversion to laparotomy, surgical blood loss, lymph node retrieval, and postoperative issues like anastomotic leakage, ileus, and wound infections. Additionally, recovery measures like first flatus time, liquid diet commencement, and post-surgery hospital stay were similar across BMI categories. These findings suggest that although higher BMI is associated with longer operative times, its impact on surgical blood loss, postoperative complications, and recovery process appears limited.

**Table 2:** Comparison of intraoperative outcomes, postoperative complications, and recovery.

Variables	Normal weight (n = 93) N (%)	Overweight (n = 36) N (%)	Obese (n = 12) N (%)	P_1_ Normal versus overweight	P_2_ Normal versus obese
Operative time (min)					
Distal gastrectomy	186.24 ± 57.28	209.43 ± 61.35	224.35 ± 61.68	0.045	0.034
Total gastrectomy	226.57 ± 65.34	252.62 ± 69.42	268.35 ± 71.22	0.048	0.042
Overall	206.22 ± 62.67	231.32 ± 66.54	246.78 ± 65.53	0.047	0.038
Conversion to laparotomy	2 (2.2)	1 (2.8)	0 (0.0)	0.832	0.623
Blood lost (mL)	103.07 ± 72.33	116.89 ± 76.37	128.54 ± 86.33	0.340	0.264
No. harvested lymph nodes	31.38 ± 12.87	29.34 ± 11.76	27.78 ± 11.53	0.410	0.359
Overall complications, n (%)	15 (16.1)	7 (19.4)	3 (25.0)	0.653	0.443
Anastomotic leakage	1 (1.1)	0 (0)	0 (0)	0.532	0.718
Anastomotic stenosis	1 (1.1)	1 (2.8)	0 (0)	0.483	0.718
Ileus	2 (2.2)	1 (2.8)	0 (0)	0.832	0.375
Wound infection	1 (1.1)	1 (2.8)	1 (8.3)	0.483	0.083
Abdominal infection	3 (3.2)	2 (5.6)	1 (8.3)	0.539	0.384
Lymphatic leak	2 (2.2)	1 (2.8)	0 (0)	0.832	0.375
Lung infection	5 (5.4)	4 (11.1)	1 (8.3)	0.251	0.678
Mortality, n (%)	0 (0)	0 (0)	0 (0)	1	1
Time to first flatus (day)	2.73 ± 1.28	2.80 ± 1.13	2.77 ± 1.21	0.774	0.918
Time to starting liquid diet (day)	3.13 ± 1.41	3.24 ± 1.36	3.22 ± 1.39	0.835	0.689
Postoperative hospital stay (day)	9.16 ± 5.34	9.62 ± 5.15	9.89 ± 5.32	0.658	0.657

Values shown are mean ± SD or n (%).

### Survival analysis

**Figure 2:**
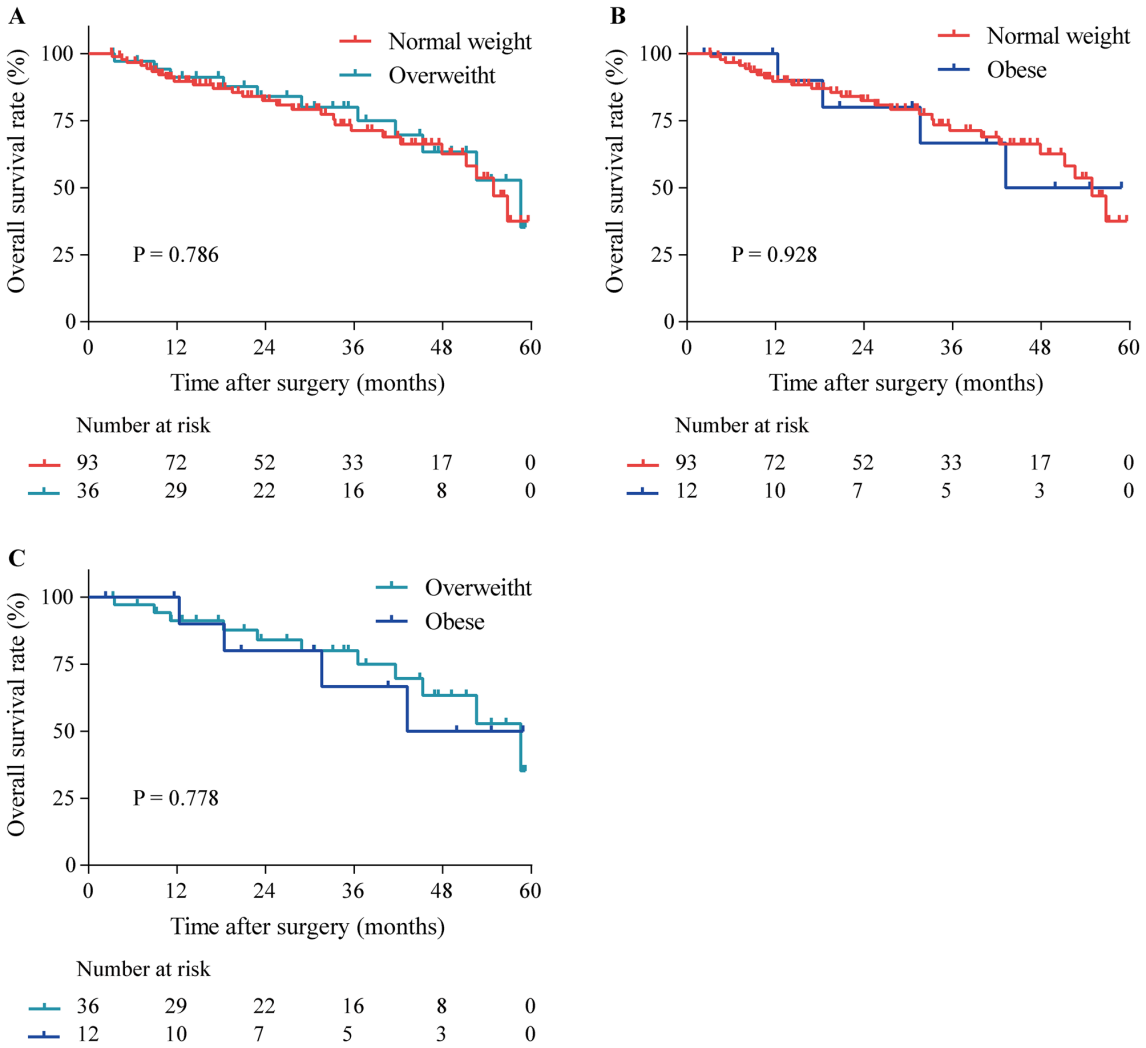
Kaplan–Meier survival curves conducted for patients, stratified by BMI: (**A**) comparison between normal weight and overweight groups, (**B**) comparison between normal weight and obese groups, and (**C**) comparison between overweight and obese groups.

Survival analysis revealed no significant difference in overall survival (OS) between overweight and normal weight groups (Fig. 2A, P = 0.786), as well as between obese and normal weight groups (Fig. 2B, P = 0.928). Additionally, the comparison of OS between overweight and obese groups revealed no significant difference (Fig. 2C, P = 0.778). This suggests that OS is consistent across normal weight, overweight, and obese groups, indicating that BMI does not significantly impact the OS of patients in this study.

### Literature review

Table 3, based on a literature review, explored the influence of BMI on short-term surgical outcomes in LG. The findings indicated discrepancies in whether high BMI prolongs operative time in LG. Of twenty studies included in our review, sixteen studies reported prolongation of operative time for elevated BMI^14–17,20,25–37^, four studies indicated a lack of significant correlation between elevated BMI and operative time^18,20,38,39^. Ten studies reported that high BMI increased the risk of more blood loss during surgery^15–17,27,28,31,32,34,36,37^. Additionally, five studies reported that elevated BMI increased the risk of postoperative complications^20,26,33,34,39^, such as intra-abdominal abscess, wound infection, lung infection, postoperative diarrhea, and subcutaneous emphysema (all P < 0.05).

**Table 3:** Effect of BMI on laparoscopic gastrectomy, based on Literature Review.

Study	Design	Country	Sample size	Type of surgery	Operative time or postoperative complications	P
Kim et al., 2011^25^	Retrospective	Korea	1100	LAG/TLG	BMI = 25.0–29.9 kg/m^2^, prolong operative time BMI = 25.0–29.9 kg/m^2^, increase postoperative complication rates BMI ≥ 30 kg/m^2^, prolong operative time BMI ≥ 30 kg/m^2^, increase postoperative complication rates	*P* < 0.001 *P* = 0.015 *P* < 0.001 *P* = 0.014
Kawamura et al., 2011^16^	Retrospective	Japan	249	LAG	BMI ≥ 30 kg/m^2^, prolong operative time BMI ≥ 30 kg/m^2^, more operative blood loss	*P* < 0.001 *P* = 0.003
Oki et al., 2012^18^	Retrospective	Japan	138	TLG	BMI ≥ 25 kg/m^2^, not prolong operative time	P = 0.790
Zhang et al., 2013^27^	Retrospective	China	114	LAG	BMI ≥ 25 kg/m^2^, prolong operative time BMI ≥ 25 kg/m^2^, more estimated blood loss	*P* = 0.030 *P* = 0.004
Sugimoto et al., 2013^28^	Retrospective	Japan	230	TLG	BMI ≥ 25 kg/m^2^, prolong operative time BMI ≥ 25 kg/m^2^, more estimated blood loss	*P* = 0.005 *P* = 0.013
Jung et al., 2014^29^	Retrospective	Korea	1,512	LAG/TLG	BMI = 25.0–29.9 kg/m^2^, prolong operative time BMI ≥ 30 kg/m^2^, prolong operative time;	*P* = 0.010 *P* < 0.001
Wang et al., 2015^14^	Retrospective	China	131	LAG	BMI ≥ 30 kg/m^2^, prolong operative time	*P* = 0.026
Lee et al., 2015^30^	Retrospective	Korea	400	RDG/TLG	BMI ≥ 25 kg/m^2^, prolong operative time	*P* = 0.001
Shin et al., 2015^38^	Retrospective	Korea	217	LAG	BMI ≥ 25 kg/m^2^, not prolong operative time	*P* = 0.827
Chen et al., 2017^31^	Retrospective	China	1691	LAG/TLG	BMI ≥ 25 kg/m^2^, prolong operative time BMI ≥ 25 kg/m^2^, more blood loss	*P* < 0.001 *P* < 0.001
Tan et al., 2017^19^	Retrospective	China	210	LAG	BMI ≥ 30 kg/m^2^, not prolong operative time BMI ≥ 30 kg/m^2^, wound infection and pneumonia	*P* = 0.128 *P* < 0.05
Shimada et al., 2018^15^	Retrospective	Japan	243	LAG	BMI ≥ 25 kg/m^2^, prolong operative time BMI ≥ 25 kg/m^2^, more estimated blood loss	*P* = 0.007 *P* = 0.007
Liu et al., 2018^20^	Retrospective	China	133	LAG	BMI ≥ 25 kg/m^2^, not prolong operative time BMI ≥ 25 kg/m^2^, increased postoperative diarrhea	*P* = 0.472 *P* = 0.027
Kim et al., 2018^32^	Prospective	Korea	1388	TLG/RDG	BMI ≥ 25 kg/m^2^, prolong operative time BMI ≥ 25 kg/m^2^, more estimated blood loss	*P* = 0.009 *P* = 0.001
Liu et al., 2019^33^	Retrospective	China	265	LAG	BMI ≥ 25 kg/m^2^, prolong operative time BMI ≥ 25 kg/m^2^, increased lung infection BMI < 25 kg/m^2^, increased subcutaneous emphysema	*P* = 0.017 *P* = 0.012 *P* = 0.025
Kashihara et al., 2021^34^	Retrospective	Japan	219	TLG	BMI ≥ 28 kg/m^2^, prolong operative time BMI ≥ 28 kg/m^2^, more blood loss; BMI ≥ 28 kg/m^2^, more postoperative complications	*P* < 0.05 *P* < 0.05 *P* < 0.05
Suematsu et al., 2022^35^	Retrospective	Japan	136	TLG	BMI ≥ 25 kg/m^2^, prolong operative time	*P* = 0.002
Maejima et al., 2022^36^	Retrospective	Japan	117	TLG	BMI ≥ 25 kg/m^2^, prolong operative time BMI ≥ 25 kg/m^2^, more estimated blood loss	*P* < 0.05 *P* < 0.05
Kim et al., 2022^37^	Retrospective	Korea	211	TLG	BMI ≥ 25 kg/m^2^, prolong operative time BMI ≥ 25 kg/m^2^, more estimated blood loss	*P* = 0.044 *P* = 0.001
Gao et al.,2022^17^	Retrospective	China	820	TLG/RDG	BMI ≥ 24 kg/m^2^, prolong operative time BMI ≥ 24 kg/m^2^, more estimated blood loss	*P* < 0.001 *P* < 0.001

LAG laparoscopy-assisted gastrectomy, TLG totally laparoscopic gastrectomy, RDG robotic distal gastrectomy ^1^.

^1^Reference^19^

## Discussion

The WHO's 2020 report labeled obesity a global epidemic and a major health crisis, underscoring its significant impact on major surgical outcomes, including those for gastric cancer^1^. Complications linked to obesity, like Type 2 diabetes, hypertension, cardiovascular diseases, obstructive sleep apnea syndrome, and non-alcoholic fatty liver disease, affect a patient's resilience to surgical stress and potential postoperative complications^7^. Moreover, the excessive adipose tissue presents additional technical challenges in surgical procedures.

The impact of obesity on LG for gastric cancer has been a focal point of numerous studies^17,20,37^, yielding contradictory results. This difference is mainly attributed to the lower prevalence of obesity in Asian countries compared to Western nations. Most studies utilize the Asia–Pacific obesity criteria, which differ from WHO standards, thereby making their findings less applicable to Western populations. Our study divided subjects into normal weight, overweight, and obese categories based on WHO BMI criteria to assess the impact of different BMIs on operative time, postoperative complications, and long-term survival in LG for gastric cancer. The results indicated a positive correlation between increased BMI and prolonged operative time, but limited impact on surgical blood loss, post-surgery complications, and recovery process. Our additional analyses showed that both distal and total gastrectomies had significantly longer operative times with higher BMI (all P < 0.05). Additionally, BMI did not show a significant effect on OS.

Extensive research consistently links increased BMI with longer operative time^15,26,30,34,36^. The primary contributor to this is the accumulation of visceral and pancreatic fat in obese patients. This excess fat obscures key anatomical planes, complicating surgical navigation and, as a result, prolonging the operation^40^. Moreover, the enlarged omentum and mesentery in these patients not only make the dissection process more challenging but also add complexity to the reconstruction phase^41^, thereby extending the operative time. Anesthesia in obese patients presents its own set of complexities^42^. The necessity for precise drug monitoring and adjustment due to their unique pharmacokinetics further adds to the overall surgery duration. Complicating matters further, positioning patients with larger body sizes and unique body shapes is both challenging and time-consuming^21^. Lastly, the need for meticulous wound closure and increased focus on infection prevention in obese patients also contributes to the prolonged duration of surgical procedures^35^. Despite these challenges, our study observed no significant differences in intraoperative blood loss or the number of lymph nodes dissected across different BMI groups. This might be attributed to our smaller sample size, yet it aligns with other studies that also found no significant disparities in these metrics between different BMI groups^14,29,39^.

Similarly, our study supports previous findings that elevated BMI is associated with a higher overall risk of postoperative complications^26,33,39^, though not to a statistically significant extent. Patients with elevated BMI exhibited a heightened susceptibility to pulmonary complications, possibly due to prolonged anesthesia and the known adverse effects of high intra-abdominal pressure on lung function^39,43^. Metabolic effects of diabetes, frequently observed as a comorbidity in patients with higher BMI, such as insulin resistance, poor glycemic control, and immunosuppression, might contribute to pulmonary complications^44^. Additionally, while there was a tendency towards increased rates of wound infection in obese patients, the difference did not reach statistical significance.

Adverse postoperative events have been linked to unfavorable long-term outcomes, including OS, although this association has been disputed in other studies^5,15–17,31,38^. Our survival analysis showed no significant differences in OS between different BMI groups, indicating that BMI does not exert a substantial effect on patient’s survival. Adverse long-term outcomes post-tumor surgery are more closely associated with tumor characteristics, disease staging, postoperative treatment, and other oncological factors, with limited impact from BMI.

Table 3 of this study compiled over a decade's worth of research on patients with high BMI undergoing LG, with a particular focus on operative time and postoperative complications. The majority of these studies indicated that a higher BMI correlates with longer operative time. A minority of studies, however, did not find this relationship, potentially due to smaller sample sizes (ranging from 133 to 217 participants) leading to lower statistical power. About half of the research suggested that a higher BMI is associated with increased intraoperative bleeding. Fewer studies linked higher BMI with postoperative complications, predominantly those related to infections. Notably, studies using totally laparoscopic gastrectomy (TLG) or robotic distal gastrectomy (RDG) consistently showed that high BMI significantly extended the duration of surgery, but did not increase the risk of postoperative complications. These findings emphasize the prolonged operative time in LG procedures for patients with high BMI, while affirming the reliability and safety of the surgery.

A potential confounding factor in our analysis is the higher proportion of total gastrectomies in the obese group. Total gastrectomy is more complex and time-consuming than distal gastrectomy due to more extensive resection and reconstruction. This complexity likely contributes to the longer operative times in the obese group. Thus, while data confirm higher BMI is associated with longer operative time, procedural distribution must be considered when interpreting these results. Future studies should match procedural types across BMI categories to clarify BMI's impact on operative time without the confounding effect of differing procedure complexity. This approach will ensure a more precise understanding of BMI's influence on surgical duration, improving surgical planning and patient counseling.

Our study offers several novel contributions to the existing body of literature on BMI and laparoscopic gastrectomy for gastric cancer. First, by focusing on a specific population from Nanchang and surrounding regions, we provide insights that may differ from other regions in mainland China. Second, the use of strict case selection criteria and standardized surgical procedures by an experienced surgical team ensures the reliability and consistency of our results. Third, our study includes a comprehensive analysis of a wide range of postoperative complications, providing a more holistic evaluation of surgical outcomes. Finally, the detailed long-term follow-up data, including OS, offer valuable insights that are relatively scarce in current research. These factors collectively underscore the originality and importance of our research, providing a foundation for future studies in this field.

Despite this study providing valuable insights into the impact of elevated BMI on LG for gastric cancer, several limitations warrant consideration. Firstly, its retrospective design might lead to selection and information biases, impacting the accuracy of the results. To mitigate this, we strictly adhered to inclusion and exclusion criteria and conducted multivariate analyses to control for potential confounding factors. Future studies should adopt prospective designs to further minimize the risk of bias. Secondly, the generalizability and external validity of the findings are limited due to the sample being sourced from a single center and the small sample size, particularly with only 12 obese patients representing less than 10% of the total sample. To address this limitation, we recommend that future research should involve multicenter collaborations and larger sample sizes to enhance the generalizability and external validity of the findings. Furthermore, the study did not thoroughly assess the specific impact of fat distribution, overlooking the potential close relationship between abdominal visceral fat and both the surgical complexity and the likelihood of experiencing complications. To manage this limitation, future studies should incorporate more detailed assessments of fat distribution, such as imaging techniques, to provide a comprehensive understanding of how fat distribution affects surgical outcomes and complications. Lastly, the research primarily focused on quantifiable indicators such as operative time, postoperative complications, and OS, without fully considering subjective indicators like postoperative quality of life, which are crucial for evaluating the success of surgery. To address this gap, we suggest that future research include postoperative quality of life assessments, such as patient-reported outcome measures (PROMs) and quality of life questionnaires, to provide a holistic evaluation of the impact of obesity on postoperative outcomes. By implementing these improvements, we hope that future studies will comprehensively explore the impact of elevated BMI on laparoscopic gastrectomy for gastric cancer and provide stronger evidence for clinical practice.

## Conclusion

This study indicated that in LG for gastric cancer, while elevated BMI correlates with extended operative time, its influence on postoperative complications is minimal. Notably, a higher BMI does not negatively affect the long-term survival of patients, underscoring the safety of LG as a treatment option for obese individuals. LG is a feasible option for obese patients with gastric cancer. Future studies should focus on developing more precise assessment tools that take into account obesity-related surgical risk factors, such as metabolic comorbidities, nutritional status of patients, and weight changes, to achieve more consistent and accurate risk assessments.

## Data Availability

The datasets used and/or analyzed during the current study are available from the corresponding author on reasonable request.
